# Digital Citizen Science for Responding to COVID-19 Crisis: Experiences from Iran

**DOI:** 10.3390/ijerph18189666

**Published:** 2021-09-14

**Authors:** Hossein Vahidi, Mohammad Taleai, Wanglin Yan, Rajib Shaw

**Affiliations:** 1 EcoGIS Lab, Graduate School of Media and Governance, Keio University, Fujisawa 252-0882, Kanagawa, Japan; yan@sfc.keio.ac.jp; 2 Spatial Decision Making & Smart Cities Lab, Faculty of Geodesy and Geomatics Engineering, K. N. Toosi University of Technology, Tehran 15433-19967, Iran; taleai@kntu.ac.ir; 3 Global Resilience Innovation Laboratory, Graduate School of Media and Governance, Keio University, Fujisawa 252-0882, Kanagawa, Japan; shaw@sfc.keio.ac.jp

**Keywords:** citizen science, crowdsourcing, coronavirus disease 2019 (COVID-19), user-generated content (UGC), volunteered geographic information (VGI), public health monitoring, public health promotion, emergency management, mobile health (mHealth), digital contact tracing

## Abstract

The Coronavirus Disease 2019 (COVID-19) pandemic has so far been the most severe global public health emergency in this century. Generally, citizen science can provide a complement to authoritative scientific practices for responding to this highly complex biological threat and its adverse consequences. Several citizen science projects have been designed and operationalized for responding to COVID-19 in Iran since the infection began. However, these projects have mostly been overlooked in the existing literature on citizen science. This research sheds light on the most significant online citizen science projects to respond to the COVID-19 crisis in Iran. Furthermore, it highlights some of the opportunities and challenges associated with the strengths and weaknesses of these projects. Moreover, this study captures and discusses some considerable insights and lessons learned from the failures and successes of these projects and provides solutions to overcome some recognized challenges and weaknesses of these projects. The outcomes of this synthesis provide potentially helpful directions for current and future citizen science projects—particularly those aiming to respond to biological disasters such as the COVID-19 pandemic.

## 1. Introduction

Coronavirus Disease 2019 (COVID-19) has become undisputedly the most severe biological hazard that has been seen in the recent past. It is difficult to track the numbers, with more than 142 million people affected in 192 countries and territories as of mid-April 2021, resulting in more than 3 million deaths and numerous sufferings that have been impacting all parts of societies [1]. This is possibly the true representation of our globalized and inter-connected world, where risk is shared globally, yet disproportionately, depending on the country and community’s vulnerability, exposures, capacities, governance mechanisms, technology advancement, innovation, and more importantly, citizen behavior. There is still a massive gap between our current state of knowledge and the knowledge we need as well as between the available resources and the resources we demand to effectively fight against COVID-19. COVID-19 is a novel type of biological hazard whose many aspects, such as biology, transmission mechanisms, spread and infection trajectory, and treatment, as well as short- and long-term physical, mental, social, and economic consequences, are often uncertain or not (fully) known. Hence, COVID-19 has raised many new questions and presented various challenges for scientists, practitioners, and policy-makers since the pandemic began. This pandemic has shown that professionals and governments are not always equipped or capable of responding to a biological disaster [2] alone, since real-time/near real-time geographically distributed data acquisition, data management, data processing and interpretation, data exchange, and information presentation are the core of its response strategy.

Citizen science is a scientific practice performed, in whole or in part, by volunteers from the general public [3]. The citizen science movement aims to connect people to science and bridge the gap between the public and scientists [4]. It empowers the general public to make a direct contribution to scientific research [5]. Public participation in scientific research often allows scientists to leverage the power of volunteers to accomplish tasks that would be too expensive or time-consuming to accomplish through other means [6]. Citizen science also offers other potential benefits to professionals and citizens. These include enabling the democratization of science, allowing the incorporation of local, traditional, or indigenous knowledge of citizens in scientific research, providing learning opportunities for citizens, raising awareness in citizens, increasing advocacy among citizens, promoting behavior change among citizens, and enhancing citizens’ physical and mental health, personal enjoyment, social interaction, and satisfaction through contributing to scientific evidence [7,8,9]. The advent of information and communication technology (ICT) and the rise of Web 2.0 [10] over the past two decades have created a wide range of opportunities for citizen science. The online digital technologies can usually ease the establishment and management of citizen science projects for professionals, and can usually simplify the processing, dissemination, and presentation of the contents produced in citizen science projects for them [11]. These technologies can also facilitate the interactions and communications among professionals and people, allow geographically dispersed people to participate in citizen science projects, ease data gathering and content generation tasks for citizens, and help volunteers to enhance the quality of their contributions [11,12,13,14].

Recently, the field of citizen science has aroused increasing interest in various areas of health and biomedical sciences such as epidemiological monitoring, health behavior surveillance, environmental health study, molecular biology, and genomics (for more information, see [15,16,17,18,19,20,21]). However, to date, relatively less contribution has been made to this area compared to well-established areas of citizen science such as ecology, conservation, earth sciences, and astronomy [22]. The application of citizen science in the different phases of disaster management has also gradually been growing over the past few years. Citizen science has been deployed for addressing various types of disasters, such as earthquakes, floods, hurricanes, biological disasters, and nuclear disasters in previous projects [23] (for more information, see [24,25,26,27,28]). The experiences gained from using citizen science in past disasters such as the 2010 Haiti earthquake [29], West African Ebola virus epidemic [30,31], and Fukushima Daiichi nuclear disaster [32,33] have shown that generally, citizen science can provide great opportunities for enhancing disaster management capabilities and for reinforcing disaster resilience [34].

Given the various benefits, citizen science can generally provide a complement to authoritative scientific practices for responding to the COVID-19 pandemic and its detrimental consequences. Since the COVID-19 outbreak began, the use of online citizen science [35] for coping with COVID-19 and its adverse effects has been adopted for a range of purposes. For instance, online citizen science has been used for identification and mapping of suspected COVID-19 cases [36], tracing close contact with positive cases of COVID-19 [37], collecting respiratory sounds to aid diagnosis of COVID-19 [38], and studying risk factors for COVID-19 infection in a large, geographically heterogeneous cohort [39]. Furthermore, it has also been deployed in other COVID-19-related areas, such as designing proteins that can be able to bind to and neutralize COVID-19 [40], analyzing the existing medicines and anti-viral food molecules to identify previously unknown COVID-19 anti-viral properties [41], monitoring emotional responses to the COVID-19 pandemic [42], and studying the long-term physical, mental, and socioeconomic impacts of the COVID-19 pandemic [43].

It is said [44] that ten years of innovation have been done within a year of the pandemic across the globe, and the same is true for Iran. While COVID-19 has brought significant pressure to the whole society, various social and technological innovations have emerged in Iran. For example, several citizen science projects have been designed and operationalized for responding to COVID-19 in Iran immediately after the infection hit the country—something that was not experienced before in the ecosystem of Iranian citizen science projects. Since Iran was one of the first countries affected by the pandemic, most of the Iranian COVID-19-related citizen science projects are among the first examples of their kind in the world. However, they have not been (well-)documented in the related literature so far, similar to the former Iranian citizen science projects. Differences in culture, language, educational system, political system, deployed instruments, adopted protocols and procedures, funding structures, etc., across the world might influence how citizen science is understood, valued, implemented, and grown [45]. Therefore, every single citizen science project usually has its own characteristics which make it unique. In this sense, the documentation of citizen science projects across the globe is imperative to understand different attitudes towards citizen science, to reflect barriers to implementation and development of projects, to capture different adopted solutions and to record experiences, knowledge, insights, and understanding to be used in future contributions. Detailed capturing and investigation of the cumulated experiences and lessons learned from failures and successes of the performed citizen science projects are essential to improve future stages of current citizen science projects and successful implementation and improvement of future citizen science projects.

The main goals of this research are (1) to highlight the most significant online citizen science projects that were carried out for responding to the COVID-19 crisis in Iran, (2) to highlight some of the opportunities and challenges associated with the strengths and weaknesses of these projects, (3) to provide solutions to overcome these weaknesses, (4) to provide lessons learned and insights from Iranian COVID-19-related projects for deploying in the current and future related citizen science projects and directing future researches in this area. This research makes interdisciplinary connections between citizen science and other domains such as emergency management, public health, GIScience, informatics, and information technology (IT) to achieve the aforementioned goals. The remainder of this paper is organized as follows: Section 2 provides a brief overview of some of the main characteristics and typologies of citizen science projects. Section 3 aims to shed light on the state of citizen science in Iran that has been overlooked in the related literature and provides a brief overview of the situation of the Iranian citizen science ecosystem where COVID-19-related citizen science projects have been grown. Section 4 presents a brief overview of the timeline and condition of COVID-19 in Iran. Section 5 reviews the state of volunteering for coping with the COVID-19 crisis in Iran. Section 6 introduces five significant COVID-19-related online citizen science projects launched by 31 December 2020 in Iran in detail. Based on these backgrounds, Section 7 presents some causes of success and failure of COVID-19-related online citizen science projects, discusses some salient insights obtained from these projects, and offers some directions for improving current and future citizen science projects—particularly those for coping with COVID-19 or tackling future pandemics. Finally, the last section is reserved for the conclusion and some recommendations for future work.

## 2. Characteristics and Typologies of Citizen Science Projects

Citizen science projects can be classified in many ways considering their various characteristics. For example, they can be categorized into two major classes based on their main outcomes: a scientific/research outcome or a science-informed management/policy/response outcome [46,47]. Citizen science projects can also be categorized based on their leadership approach. A citizen science project can have top-down or bottom-up leadership. The citizen science projects that are majorly led by scientists/researchers working in an academic or a research institution (i.e., scientist-/researcher-led citizen science project), managers/practitioners of the public sector (hereinafter referred to as public sector-led citizen science project), and staff at a civil-society organization (i.e., civil-society-led citizen science project) use a top-down leadership approach. Citizen science projects that are led by an individual (i.e., individual-led citizen science project) or a community (i.e., community-led citizen science project) use bottom-up leadership [46,47]. Categorization of citizen science activities based on the expected engagement length for the fulfillment of the task(s) in citizen science projects is also feasible. Depending on the nature of the tasks, some tasks in some citizen science projects can be fulfilled by the one-time contributions of citizens. However, to fulfill some other types of citizen science tasks, citizens are expected to engage more than once in the project. For these types of tasks, the expected engagement length can range from a short-term engagement (a few days or weeks) to long-term engagement (every day and/or over a long period of time) [46,48,49]. The stage(s) of volunteers’ involvement can also be used as a means of classification for citizen science activities. Volunteers can be involved in a single stage (including problem definition, study design, research tools/methods design, data gathering and basic processing/interpretation, data analysis and interpretation, or presentation and dissemination of results) or multiple stages of a scientific practice [46,48]. Shirk, et al. [50] divided citizen science projects depending on the degree of participation. In Shirk et al.’s developed typology [50] (hereinafter referred to as Shirk et al. typology), the citizen science projects were classified into five main categories: (1) “contractual” projects, where communities request professional researchers to perform a specific scientific investigation and report on the results; (2) “contributory” projects, which are generally designed by professionals (e.g., scientists and researchers) and for which citizens primarily contribute data; (3) “collaborative” projects, which are generally designed by professionals and for which citizens contribute data but also help to refine project design, analyze data, and/or disseminate findings; (4) “co-created” projects, which are designed by professionals and citizens working together and for which at least some of the public participants are actively engaged in most or all aspects of the research process; and (5) “collegial” contributions, where non-credentialed individuals perform research independently with varying degrees of expected recognition by professionals. Haklay [51] proposed a typology (hereinafter referred to as Haklay typology) for the classification of the levels of participation in citizen science projects. In this typology, four levels of participation in citizen science were identified: (1) “crowdsourcing”, where the citizen acts as a sensor or participates in volunteered computing, (2) “distributed intelligence”, where the citizen performs as a basic interpreter, (3) “participatory science”, where the citizen contributes to problem definition and data collection, and (4) “extreme citizen science”, which includes collaboration between the citizen and scientists in problem definition, data collection, and data analysis.

## 3. Citizen Science in Iran

The state of citizen science in Iran has not been investigated to date. In Iran, similar to many other countries [52,53], citizen science in its modern form has grown in the last decade but it is still a relatively novel and unknown concept. So far, the “Academy of Persian Language and Literature”, the official regulatory body of the Persian language in Iran, has not introduced a Persian equivalent for the term “citizen science”. However, there are several unofficial parallel translations of the term “citizen science” in Persian. Among these translations, two Persian terms of “Elm Shahrvandi” and “Daneshvari Shahrvandi” are used more frequently than others. The term “Elm Shahrvandi” literally means “the science of citizen” but may also mean “the science of citizenship” in Persian. This depends on the context in which it occurs (thus, this could sometimes be misleading if one has no background in this subject). The other more popular proposed translation, “Daneshvari Shahrvandi” literally implies “conducting the scientific action by citizen”. Overall, the term of citizen science (i.e., the different proposed translations for citizen science in Persian) is not a familiar and widely-used term in laity and academia in Iran. Moreover, currently, no organization exists in Iran to serve as a central reference point for various stakeholders of citizen science, nor to bring various stakeholders of citizen science together and create a network, to coordinate and support the citizen science landscape, and to encourage and accelerate the growth of the citizen science movement in the country. Furthermore, it is noteworthy that citizen science is not currently consolidated in national research programs in Iran.

Almost all of the relatively few existing active or completed Iranian citizen science projects have been introduced with explicitly related terminologies to citizen science (such as crowdsourcing project, participatory monitoring project, community-based monitoring project, community-based conservation project, amateur observation project, and self-reporting project) or even without referring to an explicitly related term rather than a citizen science project. Consequently, many of the participants who are involved in the Iranian citizen science projects may not even be aware that they are participating in citizen science projects. Furthermore, while the characteristics of a citizen science activity [8,47,54] can be recognized in all of these projects, the lack of a common understanding about the notion of citizen science (and its various levels and types) among the professionals has led to the underestimation and neglect of some existing Iranian citizen science projects when it comes to listing them in Iran. To the best of our knowledge, most Iranian citizen science projects were founded (or co-founded) by non-governmental organizations (NGOs) and grassroots groups, mainly unknown among the general public and their potential stakeholder groups at large. As a result, these projects have substantially remained unnoticed and undocumented in the existing literature on citizen science.

Some of the existing Iranian citizen science projects (active or completed) focus on biodiversity mapping, monitoring, and conservation (e.g., see “Iran Bird Watching” (http://www.iranbirdwatching.ir (accessed on 31 August 2021)), “IRAN-VultureConservation” (https://www.vultures.ir (accessed on 31 August 2021)), “TehranBirds” (http://tehranbirds.com (accessed on 31 August 2021)), “PersianLeopard” (http://persian-leopard.com (accessed on 31 August 2021)), “PLAN4theLAND’s Project on Monitoring of Breeding Birds in Hara Protected Area” [55], “Qeshm Island’s Community-based Hawksbill Turtle Conservation Project” [56], and “Common Swift Voluntary Conservation Project” (http://www.iranianbirdingclub.com (accessed on 31 August 2021))). Astronomy is a branch of science that has a high potential for attracting the contributions of volunteers in scientific research in Iran as over 10,000 active amateur astronomers (at different skill levels) and a higher number of astronomy and space enthusiasts live in Iran [57,58]. There are a few good examples of professional-amateur (pro-am) [59] shoulder-to-shoulder collaborations in the country, where teams of skilled amateur astronomers tightly work together with professional astronomers for conducting formal scientific activities and research (e.g., the monthly sighting of the lunar crescent and conducting of the related research for Hijri calendar purposes that has been organized by “Estehlal Headquarters” in Iran over the past three decades [60,61]). Moreover, there have been some opportunities for Iranian amateurs and enthusiasts to voluntarily contribute to the astronomy- and space-themed domestic citizen science projects based upon their level of expertise (e.g., see “Search for Impact Craters in Iran” [62], “Meteorite Hunting Project in Lut Desert of Iran” [63], “Dark Sky Map of Iran” (http://www.astromap.ir (accessed on 31 August 2021)), “Observation and Recording of Perseids Meteor Shower Project” (http://ramm.ir (accessed on 31 August 2021)), “Transit of Mercury Data Collection Project” [64], and “Crater Timings During Lunar Eclipse Project” [65]). While the aforementioned scientific domains are the focus of most of the existing Iranian citizen science projects, some examples of domestic citizen science projects can be distinguished in other disciplines, including ecosystem monitoring (“Participatory Project for Monitoring of Nowrozlo Wetland Ecosystem” [66]), crowdmapping (e.g., “Mashhad Mobile Map” (https://map.mashhad.ir accessed on 31 August 2021)), “RAYA” (https://map.tehran.ir (accessed on 31 August 2021)), and “ZARBIN Tree Mapping Project” [67]) and public health monitoring and promotion (“Blood Pressure Self-reporting Program” [68] of “Iran National Mobilization for Controlling of Hypertension”—see the Section 6 for the COVID-19-related instances of this category of domestic citizen science projects). It is noteworthy that before the COVID-19 outbreak in Iran, with the exception of an Iranian government-run crowdsourcing project of “Blood Pressure Self-reporting Program” (2019) that attracted approx. 500,000 volunteer citizens [69,70], all the searchable Iranian citizen science projects were small-sized projects (from several to less than 1000 members).

In addition to the domestic citizen science projects, Iranian citizen scientists are also contributing to global citizen science projects such as “OpenStreetMap (OSM)”, “eBird”, “iNaturalist”, “Galaxy Zoo”, and “International Astronomical Search Collaboration” (e.g., “All Iran Asteroid Search Campaign”, which has been organized in collaboration with Iran’s “Nojum Magazine” [71]), and contributing to the projects and campaigns of international organizations such as “International Meteor Organization”. It should be noted that the OSM community in Iran (https://osmiran.ir (accessed on 31 August 2021)) is very active and dynamic, and to the best of our knowledge, the OSM is the most popular long-running citizen science project among Iranian citizen scientists according to the number of daily active contributors. There are also a relatively large number of studies related to the OSM project (mostly in the domain of GIScience) from the perspective of volunteered geographic information (VGI) [72] that were conducted by Iranian universities and research institutes. These studies are very significant among other Iranian research works related to citizen science according to the volume of contribution.

## 4. COVID-19 Pandemic in Iran

The first human cases of COVID-19 were reported in Wuhan, China in December 2019 [73]. The World Health Organization (WHO) declared the COVID-19 outbreak as a Public Health Emergency of International Concern (PHEIC) on 30 January 2020 following the spread of the infection beyond the borders of China [74]. In late January 2020, Iran adopted its first specific precautionary measures against the spread of the COVID-19. These measures included checking the body temperature of passengers arriving from China at the airports [75,76] among others.

On 19 February 2020, the first cases of COVID-19 infection were confirmed and officially announced in Iran [76,77]. On the following day, the Iranian government established the National Task Force for Fighting Coronavirus—a high-level task force with the full authority to lead and coordinate the response to COVID-19 in the country [78,79]. By the end of February 2020, the COVID-19 virus spread was reported in over 60 countries and territories around the world [80]. On 1 March 2020, the total number of confirmed COVID-19 cases in Iran reached 978 (including 385 new daily confirmed cases) [81]. On the same day, following the spike in the number of positive COVID-19 cases in the Republic of Korea, Italy, Iran, and Japan, the WHO highlighted the epidemics in these countries as its greatest concern [82]. The WHO declared the COVID-19 outbreak as a pandemic on 11 March 2020 due to the rapid spread of the infection across the globe and the substantial number of cases reported worldwide [74]. Since then, the virus has continued to spread across the globe, including Iran. By 31 December 2020, a total of 1,225,143 COVID-19 cases have been confirmed, 55,223 people have died, and 988,838 people have recovered from the COVID-19 disease in Iran [83].

## 5. Civic Participation through Volunteering for Coping with COVID-19 Crisis in Iran

Civic participation [84,85] is a key principle for promoting emergency and crisis management and enhancing resilience [86,87,88]. Volunteering [86,89] as one of the pillars of civic participation can significantly contribute to the spectrum of emergency and crisis management phases, from mitigation/prevention and preparedness to response and recovery [90,91,92]. In the ongoing COVID-19 crisis, volunteers around the world have played influential roles in responding to the pandemic and mitigating its health, social and economic impacts on citizens and communities, and they have provided invaluable assistance for the formal organizations and governments [93,94,95].

The role of voluntary and charitable activities in responding to natural disasters and crises is significant and remarkable in Iran [96]. This is also the case in the current unprecedented state of public health emergency in Iran. Since the beginning of the COVID-19 outbreak, hundreds of thousands of volunteers across the country have stepped up and played a role in supporting their communities, the healthcare system, and other related public sectors. In many cases, the reasons behind volunteering in Iran (such as the participation of volunteers in different phases of emergency and crisis management) are linked to the moral, ethical, and religious values, beliefs, and motives of altruism, benevolence, compassion, humanitarianism, collectivism, cooperation, and attaining the pleasure of God (that have been widely instructed, strongly emphasized, and greatly praised in the rich Iranian-Islamic culture of the country) [96,97,98,99]. In this context, the faithful and devoted contributions of the unaffiliated (spontaneous) [86,100] and affiliated volunteers [86,101] to the health and welfare of the nation (along with the invaluable selfless, tireless, and diligent services of the Iranian healthcare professionals and practitioners from other sectors) have assisted the government in strengthening the battle against COVID-19 at a time when the country’s combat against COVID-19 has been impeded due to tremendous adverse effects of the tough sanctions against Iran [75,102,103,104]. Moreover, these dedicated voluntary efforts together with invaluable and sympathetic services of the public and private sectors have considerably helped citizens to overcome post-traumatic stresses [105] of COVID-19. They have also raised hope and promoted solidarity in society. Volunteers have been involved in various activities such as the production of protective equipment (e.g., mask, gown, and coverall); disinfection of public surfaces; providing assistive/professional services at medical centers; providing social work, chaplaincy, and spiritual care services; meal preparation and distribution; hygiene kits and items distribution; shopping; running other errands and driving for elderly and vulnerable people; serving at checkpoints; performing burial rituals; creating cultural products and artworks, donations of blood (and plasma), money, goods, and facilities; and waiving rental fees. Science, technology, and education are other areas where Iranian volunteers have made contributions since the beginning of the outbreak in the country (e.g., answering online surveys related to COVID-19 designed by researchers, participating in Iranian COVID-19 vaccines and drug clinical trials, designing open-source ventilators, disinfection devices, and low-cost mask- and glove-making machines, and educating the community on how to prevent COVID-19). In the following sections, some of the significant examples of online citizen science activities in Iran in response to COVID-19 as a form of volunteering in science will be introduced and discussed in detail.

## 6. Iranian Online Citizen Science Projects for Responding to COVID-19

Since the beginning of the COVID-19 outbreak, Iranians have contributed to various COVID-19-related domestic and global citizen science projects. By 31 December 2020, five significant domestic online projects with citizen science characteristics [8,47,54], including the “Ministry of Health and Medical Education” (MOHME) of Iran’s project for COVID-19 self-assessment and self-reporting (hereinafter referred to as CSASR project), AC19 project, Mask project, Gharbalgar COVID-19 project, and the project of “China-Iran Cooperation Group against COVID-19 Disease” (hereinafter referred to as CHIACD project) can be identified. The main thematic areas of these projects can be listed as (1) self-assessment and self-reporting of COVID-19 related symptoms (CSASR and AC19 projects), (2) digital contact tracing (Mask project), (3) COVID-19 cough audio sample collection (Gharbalgar COVID-19 project), and (4) translation of COVID-19 educational, scientific, and technical materials for responding to COVID-19 (CHIACD project). Three of these identified projects (CSASR, AC19, Gharbalgar COVID-19) can be categorized as contributory (crowdsourcing) citizen science projects, and the rest (Mask and CHIACD) are collegial (extreme) citizen science projects (for more information, see Section 6.1, Section 6.2, Section 6.3 and Section 6.4). As these five Iranian COVID-19-related online citizen science projects have remained unnoticed and undocumented in the existing literature in this area, their specifications and characteristics will be presented, classified, and discussed in Section 6.1, Section 6.2, Section 6.3 and Section 6.4.

### 6.1. COVID-19 Self-Assessment and Self-Reporting System and Application (App)

The CSASR project—an online project for COVID-19 self-assessment and self-reporting—was launched by MOHME of Iran [106] (https://self.salamat.gov.ir (accessed on 31 August 2021)) on 4 March 2020 under the schemes of the “Iran National Mobilization Against COVID-19” program [107]. Iranian citizens can participate in this nationwide surveillance project on a voluntary basis. To use this online web tool that is linked to the electronic health records (EHRs) of Iranians, a volunteer participant is required to provide his/her national identity number, date of birth (for validation of the identity), place of residence (province and county), and mobile phone number. By answering a few questions about the symptoms and risk factors, this tool helps the participant to voluntarily assess and report his/her symptoms. This tool, then, determines whether the participant should visit a healthcare center for further assessment and testing. The identified suspected cases of COVID-19 through this web tool are provided with advice about personal and familial protection and referred to the nearest selected health center. The participants are enabled to update and re-submit their health condition and symptom information anytime in this system. The identified suspected cases of COVID-19 through this system are contacted by healthcare workers so that further investigations can be conducted, and contact tracing can be undertaken [106] under the “Iran National Mobilization Against COVID-19” program [107]. The crowdsourced data through this symptom tracking [106] system provided the public health officials with a clearer picture of the spatio-temporal spread of COVID-19 across the country and allowed them to implement a data-driven response in the early stages of the outbreak. This participatory system enables authorities to perform early screening of citizens and triage suspected cases. Moreover, by reducing unnecessary visits to medical centers, this web tool has decreased the risk of citizens’ exposure to the disease, prevented more spread of infection, and reduced the burden on the healthcare system. Based on the latest publicly available information, by 3 April 2020, over 12 million people self-reported their symptoms and health condition via this system [108]. The number of participants in CSASR project is the highest number ever reported (by 31 December 2020) in an Iranian citizen science project (for more information, see Section 3 and Table 1). The general specifications of the CSASR project (including the project’s scientific area, the number of contributors, the least expected engagement length needed for the fulfillment of the project’s task, the adopted technology for data collection, and the launch date) were summarized and presented in Table 1.

Some of the main characteristics of the CSASR project (including the outcome of the project, the project’s leadership approach, the stage of volunteers’ involvement in the project, the type of project based on Shirk et al. typology, and the type of project based on Haklay typology) were categorized based on the presented typologies in Section 2 and were presented in Table 2.

In addition to the aforementioned main official system designed for self-assessment, self-reporting, and monitoring of COVID-19 in Iran, the “COVID-19 Disease Operation Management Headquarters in Tehran Metropolis” in collaboration with the “Ministry of Information and Communications Technology (MICT) of Iran” set up another government-led mobile (for Android platforms) and web app named “AC19” (https://ac19.ir (accessed on 31 August 2021)) on 3 March 2020 for COVID-19 self-assessment. To register in this app, the volunteers need a mobile phone number. The app asks users for their personal and biometric information (name/username, city of residence, gender, age, weight, and height). Next, users answer a series of questions about their symptoms, past medical history and underlying disease, and exposure history to evaluate whether they should visit a healthcare provider or physician for further COVID-19 related assessment. The app also gives some recommendations on what to do next. The users can repeat this online self-assessment test anytime and update their information in this app. The app also offers other services for users, such as providing the latest COVID-19 news, updates and advice from reliable sources, governmental agencies, and medical centers’ information. Contrary to the current version of the app that only records the user’s city of residence, the app had collected the precise location of the user via GPS in addition to the information about the user’s city of residence (that was directly provided by the user) in the early releases of this app—the functionality that had enabled the initiative to obtain VGI about the distribution of suspected COVID-19 cases at a fine spatial scale beside the coarse spatial scale. Iran’s minister of MICT revealed a fine-scale COVID-19 aggregate risk map for the Greater Tehran area [109] and a coarse-scale COVID-19 aggregate risk map for Iran [110] to the public using the crowdsourced information through the AC19 app on 9 March 2020 and 11 March 2020, respectively. The acquired crowdsourced information from this app was used by the officials, majorly in the first stages of the pandemic in Iran. In this term, the gathered information by this app served as a complementary data source for conducting a rough estimate of COVID-19 distribution and size of the outbreak in Tehran and across the country. It also helped raise the awareness of the public about the spread of the infection, which convinced citizens to stay at home. According to the latest publicly available information, by 15 March 2020, approx. 4 million volunteers contributed to this system [111]. The general specifications of the AC19 project (including the project’s scientific area, the number of contributors, the least expected engagement length needed for the fulfillment of the project’s task, the adopted technology for data collection, and the launch date) were summarized and presented in Table 1. Moreover, some of the main characteristics of the AC19 project (including the outcome of the project, the project’s leadership approach, the stage of volunteers’ involvement in the project, the type of project based on Shirk et al. typology, and the type of project based on Haklay typology) were categorized based on the presented typologies in Section 2 and were presented in Table 2.

### 6.2. COVID-19 Digital Contact Tracing App

The “Mask” app (https://mask.ir (accessed on 30 June 2021)) was initially designed for digital contact tracing (also known as proximity tracing or app-based contact tracing) [106,112] based on a centralized architecture [113]. This app was voluntarily developed by a group of professors, students, and graduates in the fields of Computer Engineering and Mathematical Sciences from Iranian universities (including “Sharif University of Technology”, “Shahid Beheshti University”, and “Amirkabir University of Technology”) in collaboration with a group of medical specialists in response to the outbreak of COVID-19 in their country. This non-profit project was launched on 2 March 2020 and was later endorsed and supported by MOHME of Iran [114]. Citizens’ participation in this contact tracing project was on a voluntary basis through the released mobile (for Android platforms) or web versions of the app. Once the app was downloaded or delivered over the internet through a browser interface, the users were required to register in the system using an Iranian mobile phone number and provide some information on their health condition to benefit from all the functionalities of the app. The project could log memory of all close proximity contacts of the app users using Bluetooth, GPS, manual pinning of the contact location, or scanning of barcode and QR code. The users could also record the close physical contact information of their family members using this app. The app allowed users to be informed if in the past 14 days they were closely exposed to any other app users who had been definitely diagnosed with COVID-19 by MOHME’s medical centers without revealing the identity of the infected person. Furthermore, a COVID-19 symptom checker tool for self-reporting of the daily health condition of the app users was integrated in the app. This self-evaluation function told the app users what to do next and if they needed to get medical care by asking a few questions on their health condition and symptoms and by deploying the records of the users. Besides the aforementioned major services of this app that were only available for the registered users, the app provided an interactive grid heat map of the confirmed and suspicious COVID-19 cases across Iran (produced based on the obtained data from Iran’s MOHME) and daily statistics, news, and educational and awareness materials about the COVID-19 disease that were open to the public [115,116,117]. Based on the latest publicly available information, on 21 April 2020, the total number of active installed instances of this contact tracing app (downloaded from different Android marketplaces or the project’s website) exceeded 1 million installations (for more information, see Section 7.3.1.1) [118]. Due to the relatively low uptake of this app (which has also has been a challenging issue for many similar projects worldwide—for more information, see Section 7.3.1), the Mask project stopped its digital contact tracing service in early November 2020. The general specifications of the Mask project (including the project’s scientific area, the number of registered users, the least expected engagement length needed for the fulfillment of the project’s task, the adopted technology for data collection, and the launch date) were summarized and presented in Table 1. Furthermore, some of the main characteristics of the Mask project (including the outcome of the project, the project’s leadership approach, the stages of volunteers’ involvement in the project, the type of project based on Shirk et al. typology, and the type of project based on Haklay typology) were categorized based on the presented typologies in Section 2 and were presented in Table 2.

### 6.3. COVID-19 Cough Audio Sample Collection

The “Machine Intelligence and Robotics Department, University of Tehran”, Iran launched a crowdsourcing program named “Gharbalgar COVID-19” [119] on 10 April 2020 for collecting cough sounds as well as the basic demographics and medical history of the participants. The crowdsourced data through this app is used by the researchers of this department for the training of machine learning-based predictive models that are developed for the early screening and diagnosis of suspicious and positive COVID-19 cases. Both healthy and unhealthy participants were welcomed in this project. To collect the data from the volunteers in this program, a mobile app was released for Android devices (available in Persian and Arabic languages) and a web app was developed (available in the Persian language) for the devices using other operating systems. The data was gathered from app users by utilizing a basic questionnaire and recording a few seconds of coughing through the microphone of the device [119,120]. By 28 April 2020, almost 500 volunteers contributed to the Gharbalgar COVID-19 project. The general specifications of the Gharbalgar COVID-19 project (including the project’s scientific area, the number of contributors, the least expected engagement length needed for the fulfillment of the project’s task, the adopted technology for data collection, and the launch date) were summarized and presented in Table 1. Moreover, some of the main characteristics of the Gharbalgar COVID-19 project (including the outcome of the project, the project’s leadership approach, the stage of volunteers’ involvement in the project, the type of project based on Shirk et al. typology, and the type of project based on Haklay typology) were categorized based on the presented typologies in Section 2 and were presented in Table 2.

### 6.4. Translation of COVID-19 Educational, Scientific, and Technical Materials

Initiated by a Chinese language instructor, the CHIACD project [121] was established on 24 February 2020. The project was established at the early stages of the pandemic when the appropriate and reliable resources about prevention and protection methods against COVID-19 in languages other than Chinese were limited. Thus, this project aims to raise awareness and educate the Iranians about the COVID-19 disease by voluntary gathering, synthesizing, and translating the Chinese educational and scientific materials about COVID-19, presenting the produced outputs in appropriate formats, and disseminating them in society. Moreover, it aims to transfer China’s experiences in fighting COVID-19 to the Iranian health workers through voluntary translation and dissemination of the Chinese educational, scientific, and technical materials about COVID-19 [122]. The first core members of this voluntary group were the Chinese and Persian language students and scholars from Iran and China. Later, new volunteers with different backgrounds (e.g., medical sciences and engineering), nationalities (Afghan volunteers), and Chinese and Persian language familiarity levels have joined this team over time. To put various capabilities and the expertise of the volunteers to better use, and to facilitate the collaboration and drive the group’s objectives, the volunteers’ activities have been structured under the following working groups: (1) information collection, (2) general translation, (3) medical translation, (4) external relations with the health workers and centers, (5) graphic design, (6) video production, (7) proofreading and quality assurance, and (8) digital products dissemination. The group mainly uses WeChat, a messaging and social media app to organize the group’s activities, hold meetings, facilitate the communication between members and working groups, assign tasks, and exchange raw and produced digital materials. The group is obtaining COVID-19 related information only from authoritative and reliable Chinese sources. Public translation services that can be performed by a translator’s general knowledge are mostly conducted by volunteer translators from fields other than medical sciences in this project. To enhance the quality of the medical specialized translation tasks, these translations are only performed by or under the direct supervision of the volunteer translators with a medical science background. Furthermore, before the dissemination of the group’s translated products to the audience of the project, all the products are reviewed carefully by a team volunteers to ensure the appropriate quality of the final products. The generated contents of the group are shared on official pages and groups of the project on social media and messaging platforms (Instagram, Aparat, Telegram, and Twitter) for the general public audience of the group. In addition to the group’s commitment to raising the awareness of the citizens about the COVID-19 disease, the group has been able to play a role in creating a direct bridge between Iranian and Chinese health workers and medical centers for exchanging their latest scientific information and experiences alongside the existing formal intergovernmental collaborations. In this term, the group is voluntarily collaborating with an Iranian social network for medical science professionals named “TritApp” (https://tritapp.net (accessed on 31 August 2021)) for establishing the communications and translation of the exchanged Persian and Chinese questions and answers between Iranian and Chinese health workers and medical centers in the two countries [123]. In early May 2020, the total number of volunteer members in the CHIACD project was around 300. The general specifications of the CHIACD project (including the project’s scientific area, the number of contributors, the least expected engagement length needed for the fulfillment of the project’s tasks, the adopted technology for data collection/data exchange, and the launch date) were summarized and presented in Table 1. Furthermore, some of the main characteristics of the CHIACD project (including the outcome of the project, the project’s leadership approach, the stages of volunteers’ involvement in the project, the type of project based on Shirk et al. typology, and the type of project based on Haklay typology) were categorized based on the presented typologies in Section 2 and were presented in Table 2.

## 7. Insights from the Past for the Current and Future COVID-19-Related Citizen Science Projects

In the following sections, some insights from five selected Iranian COVID-19-related citizen science projects as well as other related studies in this area will be presented and discussed to be used in citizen science projects in the future, particularly those in the domain of biological disasters such as COVID-19.

### 7.1. Initial Steps Needed for Capacity Building for Citizen Science in Public Sector and Academic and Research Institutions of Iran

To build the capacity for citizen science projects, five critical steps can be recognized: (1) identifying and engaging all actors in the citizen science projects, and enhancing the visibility of citizen science activities, (2) assessing the capabilities and needs of stakeholders, (3) developing citizen science projects’ visions, missions, and action framework, (4) developing resources (e.g., developing technical infrastructure, guidelines, and educational material and provision of financial and human resources), and (5) evaluating the implementation to foster further development [124]. Citizen science capacity building is an iterative and adaptive mechanism that requires the active engagement of all stakeholders from society, science, and policy [124].

In Iran, the government has a significant influence on social, economic, and technological changes. As discussed earlier, citizen science is a relatively new concept in Iran. Currently, citizen science is not integrated into the organizational culture of governmental organizations in Iran. Thus, most of the influential people in governmental organizations who can benefit from citizen science might be unfamiliar and lack the necessary knowledge regarding how this approach can be leveraged to address various challenges encountered by their organization. Furthermore, similar to many other countries, there is still considerable skepticism among the Iranian policy-makers, decision-makers, and practitioners on the quality and trustworthiness [125] of the user-generated content (UGC) [126] produced in the citizen science projects; consequently, even many of those who are familiar with the concept usually have hesitations using such data in formal applications. Raising the profile of citizen science within an organization usually rests on the leadership of senior staff. Hence, before citizens are expected to embrace the citizen science projects, one of the vital tasks for building the capacity for government-driven citizen science projects in Iran is to raise the knowledge and come to a common understanding among the key personnel in the governmental organizations (particularly those whose involvement in and support from the ecosystem of citizen science projects is essential) about this concept, its pros and cons, and its potential applications for attaining the missions of their organizations and distributed problem-solving. Furthermore, it is necessary to change the conventional mindset of the official stakeholders of citizen science about the quality of citizen scientists’ generated content—an attitude that always underestimates the citizen science data quality. The previous studies showed that the quality of generated data in citizen science projects can approach and even may exceed that of authoritative sources [127,128]. Therefore, to deal with the existing concerns among officials, it is crucial to highlight some successful examples of citizen science projects across the globe whose outputs have been employed for solving real-world problems, and it is necessary to raise their knowledge about the existing adopted approaches for assuring and enhancing the quality of the contributions in citizen science programs. As it was mentioned before, there is currently no governmental or non-governmental organization in charge of supporting, developing, and coordinating citizen science activities in Iran. As one of the first steps, it is therefore crucial to establish a governmental entity for promoting and coordinating the utilization of citizen science across the Iranian government and society. This entity has to train human resources, provide consultation services, finance citizen science activities, provide technical and legal support, regulate and standardize citizen science activities, connect various stakeholders, and develop best practices for designing, implementing, and assessing citizen science programs in order to surmount the existing obstacles, limitations, and delays and to build the capacity for citizen science.

Citizen science can permit academic and research institutions to access to previously inaccessible areas by enriching and enlarging their research scope, can consolidate the position and recognition of academic and research institutions in society, can provide new resources and opportunities to them, and can enhance public trust in them [129]. Academic and research institutions can contribute to citizen science by providing their professional infrastructure and facilities, educational and research capabilities, ethical and legal background, and funding [129]. While in many countries, citizen science was established and mainly has been developed by academic and research bodies [130], the role of Iranian academic and research institutions in running citizen science projects has not been prominent yet. To the best of our knowledge, the most notable Iranian citizen science projects were initiated from outside of academia and research bodies. The main outcomes of notable Iranian citizen science projects (for more information, see Section 3) are mostly science-informed management, policy, or response. Citizen science projects with scientific/research outcomes in the list of notable Iranian citizen science projects are in the minority. The concept of citizen science is less well-known in most Iranian academic and research institutions. Furthermore, citizen science has not been included in the academic and research culture of Iranian academic and research institutions yet. Currently, there is no institutional structure or venue dedicated to leading, coordinating, promoting, supporting, and funding citizen science (or Open Science [131] as a related concept) within the “Ministry of Science Research and Technology” (MSRT) of Iran, MOHME of Iran, or Iranian academic and research institutions. Nowadays, many scientists and researchers across the world have been approached to design more inclusive citizen science practices to involve diverse groups of citizens in various aspects of the scientific and research process. However, in the landscape of Iranian scientist-/researcher-led citizen science projects, to date, the designed participation level for the involvement of citizens in the implemented citizen science projects has not gone beyond the crowdsourcing level, mostly due to a lack of knowledge and understanding about the concept and various aspects of citizen science. These are also the case in the context of Iranian COVID-19-related citizen science projects. Among the five identified Iranian COVID-19-related online citizen science projects, only one project (Gharbalgar COVID-19) was initiated by scientists/researchers. Similar to other Iranian scientist-/researcher-led citizen science projects, the level of citizens’ participation in this project was limited to the crowdsourcing level (Table 2).

The aforementioned recommendations for building the capacity for citizen science within the Iranian government are also generally valid for citizen science capacity building in Iranian academic and research institutions. Establishment of the institutional structures and entities in charge of leading, developing strategies and operating framework, coordinating, promoting, providing technical and legal supports, and funding citizen science within Iran’s academic and research system at different levels should be considered as a high priority task. Organizing scientific training programs and events for raising awareness, reaching a common understanding, and fostering knowledge and skills of Iranian scientists and researchers about various theoretical and practical aspects of citizen science is pivotal in shaping the citizen science movement in academic and research institutions. Consequently, it should also be given higher priority. Furthermore, as another initial step for building the capacity for citizen science and accelerating citizen science movement within Iranian academic and research institutions, it is crucial to develop free customizable platforms by (for) Iranian academic and research bodies. This will ease conducting a variety of citizen science projects by scientists and researchers.

### 7.2. Enhancing the Effectiveness of Citizen Science Projects for Emergency Response through Early Preparedness and Coordination

Contrary to many other thematic categories of citizen science, citizen science projects relevant to emergency response must become operational in a short time since deferring the issue can cause loss of life and other costs to societies and nations. Hence, to foster the effectiveness of citizen science projects for emergency response, it is crucial to build the capacity for these projects, mainly during the disaster preparedness phase. Although, in general, citizen science-based solutions have still not been embedded in the normal working routines of Iranian organizations (including the MOHME of Iran) and the organizations do not have clear and long-term visions and comprehensive plans and strategies in this area, the MOHME of Iran unveiled its COVID-19 self-assessment and self-reporting system and integrated it into its official screening and follow-up system and EHR database within a relatively short time after confirming the first cases of COVID-19 in the country. This unprecedented rapid digital response was mainly powered by the accumulated data, knowledge, and skills, and created an organizational structure and established IT infrastructure in MOHME of Iran during its prior crowdsourcing project, the Blood Pressure Self-reporting Program in 2019, which was Iran’s first large-scale experience in employing citizen science in healthcare. The previous studies [106,132,133] also showed that the pre-established organizational structures, organizational memory, pre-defined objectives, protocols, procedures and strategies, and pre-existing generic and scalable citizen science infrastructures can significantly reduce the time and cost of implementation and can enhance the organizational adaptability and applicability of citizen science programs for responding to disasters and emergencies.

The COVID-19 pandemic is the first biological disaster experience at this scale in Iran. Therefore, similar to many other countries, many loopholes and shortcomings (e.g., see [134]) emerged in the activities of the public health emergency response system in the initial phase of the epidemic in the country. In the early days of the COVID-19 outbreak in Iran, there have been simultaneous efforts (the public sector-led projects of CSASR and AC19 and the community-led project of Mask) for crowdsourcing various health information for means of epidemiological monitoring (Table 1 and Table 2). There is considerable overlap between the goals and missions of two government-led projects (CSASR and AC19)—therefore, the contents of the gathered data in these two projects were almost the same. Furthermore, there are some areas that both Mask project and the two aforementioned government-led projects are commonly collecting data about (for more information, see Section 6.1 and Section 6.2). While each of these three citizen science projects may perform well within its own functional areas, the overlaps or some similarities among the goals and missions of these projects have at best caused the fragmentation of crowdsourced health information and have at worst also caused the occurrence of duplications or redundancies in the gathered information, which is a waste of time, energy, and resources for the stakeholders and the volunteers. To reduce/eliminate the redundant data production efforts in the government-led citizen science programs, governments have to define/revise their data gathering policies, strategies, and procedures, as well as their collaboration plans in such a way to clarify and assign the roles and responsibilities of each stakeholder of citizen science projects for responding to the public health crisis in advance. Moreover, to reduce/eliminate the duplication and fragmentation of the multi-sector health information (including crowdsourced and authoritative health information) generated in the different phases of the public health crisis and to share the generated contents between the authorized stakeholders, it is necessary to aggregate and exchange the sparse datasets in compliance with the data privacy laws, protection standards, and protocols [18,135,136] in a real-time (or near real-time) manner through an integrated information system [137], data clearinghouse [138], or geoportal [139]. In this context, adopting the pre-approved protocols and standards [140] provides an effective solution for data interoperability [141] and facilitates the seamless integration of the generated crowdsourced datasets. It is noteworthy that the provision of these arrangements (which facilitates multi-sector citizen science data exchanging and data integration) may also increase the role of accredited non-government-led citizen science projects (i.e., the projects led by private research bodies and private academic institutions, civil societies, and communities) in gathering data (such as public health- and emergency-related data), which have been mostly crowdsourced so far by the governments. The inclusion of accredited non-governmental citizen science initiatives in the focused domain may trigger more social and technological innovations in data crowdsourcing that lead to providing more diversified options for the citizens (to opt into the project that best fits their requirements and preferences). Furthermore, this inclusion may facilitate the connection with people and communities and may foster the sense of trust among citizens as data contributors (by involving the non-governmental bodies in the administrative process of data gathering). Consequently, these may increase the number of data contributors in the focused domain.

### 7.3. Organizational Measures for Enhancing the Recruitment and Retention of Volunteers

Volunteers are one of the main pillars in all citizen science projects. In this sense, generally, recruiting and retaining a higher numbers of volunteer participants can accelerate the achievements of most citizen science projects and is integral to the success of many of them [142]. The recruitment and retention of citizen scientists in citizen science projects needs some organizational structures and measures. However, in Iran, which has less history in the modern form of citizen science, most citizen science project organizers generally have not had any prior training and experience in setting up and managing these projects. Moreover, as mentioned earlier, there are no scientific or professional institutions for guiding and training Iranian citizen science project organizers and transferring good practices to them. Therefore, even many of conventional organizational measures that have been widely practiced in modern citizen science projects for improving the initial participation and long-term engagement of citizen scientists have not been (fully-) implemented in most Iranian citizen projects.

The figures on the number of contributors/registered users in the identified Iranian COVID-19-related citizen science projects (Table 1) indicate that the three identified projects in the area of epidemiological monitoring (CSASR, AC19, and Mask) could attract vast populations of people to themselves. This level of public attention (in terms of the number of contributors/registered users) to Iranian citizen science projects was not experienced before in the ecosystem of Iranian citizen science projects. Engagement in CSASR and AC19 projects have a mostly one-time nature. However, for the proper fulfillment of the contact tracing task in the Mask project, a long-term engagement of the participants is necessary (Table 2). Compared with many other citizen science projects, the issues of recruitment and retaining of volunteers play a more critical and decisive role in the degree of failure and success of the digital contact tracing projects (for more information, see Section 7.3.1). This feature makes contact tracing projects good choices for studying the impact of various influencing factors on the initial participation and sustained participation of the people in the citizen science projects and creates a common ground for discussing and synthesizing the insights that can be obtained from previous related researches and practical experiences inside and outside the field of citizen science.

In this sense, in the following sections, some lessons learned from the Iranian contact tracing project about recruitment as well as some generic directions obtained from other related citizen science projects will be presented and discussed. These highlighted issues and directions might be deployed in the future not only for enhancing the recruitment and retention of volunteers in the citizen science projects for tracing infectious diseases such as COVID-19 but also in other categories of citizen science projects.

#### 7.3.1. Volunteer Recruitment and Retention in Contact Tracing Projects

Previous studies showed that voluntary digital contact tracing could reduce the spread of the COVID-19 epidemic [143,144]. However, a digital contact tracing project can be a game-changer only if it can attract and sustain the citizens’ mass engagement [143,145,146]. A study [147] in the UK estimated that the COVID-19 epidemic could be completely suppressed if at least 80% of all smartphone users (56% of the total population) adopt the digital contact tracing app. Hence, while there were early hopes that the digital contact tracing projects could play a key role in controlling COVID-19 infection in the affected countries, due to the unfulfillment of the required mass public engagement in these projects, there is no single country to date that can claim that its digital contact tracing project has significantly contributed to the reduction of COVID-19 transmission [148]. For instance, a report [149] from Iceland, a country that has had the largest uptake of a digital contact tracing app in the world (38% of the country’s population as of May 2020), indicates that the impact of digital contact tracing on reducing COVID-19 spread has not been significant yet. Another example is France’s digital contact tracing app that was initially downloaded by about 1.9 million people (approx. 2.8% of the country’s total population) as of June 2020; however, shortly after, around 0.5 million of those 1.9 million people uninstalled or deactivated the app. Until late June 2020, only 68 users of this app reported that they had been diagnosed with COVID-19; these reports resulted in notifications for only 14 other app users who had contacted these people [150]. The situation in Iran is also more or less the same. Iran was one of the pioneers in the launching of the digital contact tracing app (Mask app). The country has a young population—out of the approx. 83 million people living in Iran in 2019, 24.6% of the country’s population was under the age of 15, 22% of the population was 15–29 years old, and only 6.4% of Iran’s population was 64 years old and above [151]. Furthermore, according to a conservative estimate [152] in 2019, Iran had a considerable smartphone penetration rate of approx. 55% (for other estimates, see [153,154]). Moreover, the internet penetration rate in Iran stood at 70% by the end of 2018 [155]. However, according to available statistics, as of early June 2020 (more than 3 months after the start of the Mask project), the app had only about 1 million users (≈approx. 1.2 % of the country’s population) in Iran [156]. Even if it is ideally assumed that all the people who activated the Mask app on their smartphones actively used the main functionality of the app—contact tracing (which is a very unrealistic assumption), similar to most other existing COVID-19 contact tracing apps, the uptake rate of this app was well below the estimated threshold needed for a digital contact tracing app to be significantly effective.

Prior to the onset of the pandemic, various studies had been conducted to identify the antecedent factors for uptake and engagement with apps such as participatory mobile health (mHealth) apps (for more details, see [157,158,159]). Within the growing field of citizen science, some studies also have been conducted to determine what factors influence people’s participation in citizen science projects and why volunteers continue their participation in these projects. These studies intended to shed light on the various dimensions such as social, behavioral, cognitive, motivational, legal, managerial, technical, and technological dimensions affecting the participation and engagement of people in citizen science projects. The various influencing factors on the initial participation and sustained participation of people in the citizen science projects generally can be categorized into two broad categories of dispositional factors (i.e., attributes of individuals) and organizational factors (i.e., attributes of the project and its organizers) [142,160,161].

Most of the recent studies that have raised the problem of COVID-19 contact tracing apps’ low adoption from the organizational perspective have placed great emphasis on studying the impact of various technical factors and technology-related legal and ethical factors such as users’ privacy, data protection, transparency, service quality, instrument and interface design on the participation and long-term engagement of people in contact tracing projects. Generally, the successful addressing of technical issues and technology-related legal and ethical issues that were extensively studied and discussed in these studies (which were mostly conducted in western societies and have backgrounds other than citizen science) may lead to an increase in the adoption and user retention in the COVID-19 contact tracing apps. However, there are also some other less-discussed organizational factors beyond these factors that significantly impact the uptake and long-term deployment of contact tracing apps. To this end, in the following sections, the role of promotion, advertising, and marketing, as well as recognition, communication, and feedback on the recruitment and retention of volunteers in the contact tracing project will be discussed in more detail through forging interdisciplinary connections among citizen science, psychology, sociology, emergency management, and public health.

##### 7.3.1.1. Promotion, Advertising, and Marketing

Despite their considerable benefits, contact tracing apps are characterized by various technical shortcomings and inconveniences as well as legal and ethical concerns. Thus, if one became aware of the existence of a contact tracing project, he/she would usually estimate the perceived costs (including the various shortcomings, inconveniences, and concerns) and benefits (including personal and public benefits) of deploying a contact tracing app to decide whether to install (or continue to deploy) a contact tracing app. If the benefits of the decision outweigh its costs, the person adopts the app (or maintains its use) [162,163,164]. To ensure mass adoption and continued use of a contact tracing app, in addition to the necessity for minimizing the costs of participation in a contact tracing project (through addressing the existing shortcomings, inconveniences, and concerns) and the need for informing people about the launch of the contact tracing program, citizens must be made aware of the various benefits of attending a digital contact tracing project [163,165,166], the contents of the tasks and the updates from the project. It was argued that the lack of awareness of an existing citizen science project (i.e., volunteering opportunity) and lack of information and understanding of volunteering task(s), volunteering responsibilities, participation requirements, and personal and public benefits of the project are key barriers to recruiting people with diverse backgrounds in a citizen science project [142,167]. This implies that the promotion, advertising, and marketing for citizen science projects are effective ways for recruiting volunteers in citizen science projects and stimulating the uptake of citizen science project apps by informing people and raising public awareness and understanding [142,168]. The insufficient promotion and advertising or use of inefficient marketing strategies are among the most significant reasons for the failure of many Iranian citizen science projects in attracting citizens, and the Mask project is not an exception to this.

Contrary to most of the existing COVID-19 contact tracing apps in the world that were implemented or funded by governments, the well-developed Mask app was produced on a non-governmental, non-profit, and voluntary basis by a group of computer science and engineering scholars and experts. Consequently, the project organizers had intrinsically limited resources and authority for mounting mass nationwide advertising campaigns compared with many other similar projects around the world. The project was initially introduced by the advertisement contents (e.g., text, image, and video advertisements) shared through the project’s website, official channels on social media and messaging platforms (Telegram Messenger, Instagram, Twitter, Soroush Messenger, and Bale Messenger), and an online video-sharing platform (Aparat), that in total, these channels have been able to attract approx. 25,000 members and followers so far. The project also used celebrity and elite endorsement on social media in the early weeks of its launch. Furthermore, the project has received media coverage by various online Iranian news agencies as well as online and print newspapers since its appearance. Following the scientific and ethical approval of the project by MOHME, the MOHME and several high-ranking state officials have also formally recommended installing the Mask app or have talked about it. Moreover, the Islamic Republic of Iran Broadcasting (IRIB)—Iran’s state-owned media corporation (which owns over 70 television channels and 80 radio stations) —has covered and introduced the Mask project in several television programs.

The list of television programs related to the Mask project that were broadcasted by IRIB, as well as the formal endorsement, recommendation, and promotion of the Mask project by the MOHME and several high-ranking Iranian state officials from 2 March to 30 June 2020, were extracted from the accessible online sources. It should be noted that this list may miss a few other incidents (e.g., the Mask project’s few broadcasted short video advertisements on the IRIB television service) whose details (i.e., the incident or the occurrence date of it) could not be retrieved from available online sources. A total of 13 different incidents were identified on 9 different days in the abovementioned period, the details of which are demonstrated in Table 3.

**Figure 1:**
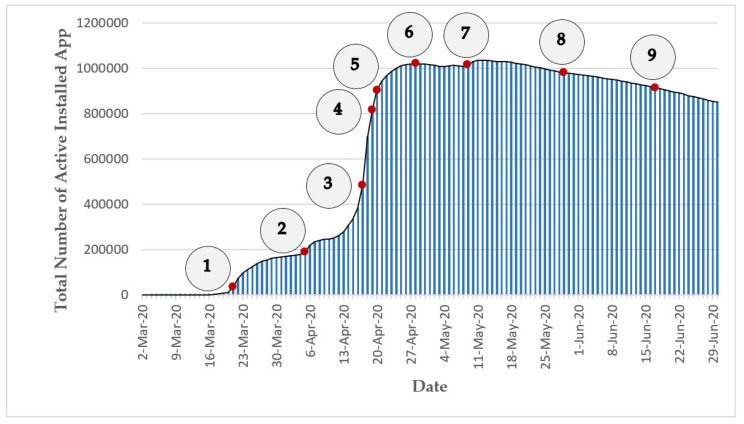
(1) Total number of active installed Mask apps per day (raw data courtesy of Mask project); (2) timeline of the identified television programs broadcasted by IRIB related to the Mask project and the endorsement, recommendation, or promotion of the Mask project by the government (from 2 March to 30 June 2020). Notes: the figures for the total number of active installed Mask apps per day are limited to the installed instances of the Mask app downloaded from Cafe Bazaar Android marketplace; the numbering of the events is the same as that in Table 3.

The Android version of Mask app (that is the most popular version of the app) can be downloaded either from Iranian Android marketplaces (Cafe Bazaar, Myket, Charkhoneh) or from the project’s website. Among these, Cafe Bazaar is by far the most popular platform for downloading this app. Figure 1 shows the “total number of active installed instances of the Mask app (downloaded from Cafe Bazaar Android marketplace)” (TNAIIMA) per day from 2 March to 30 June 2020. The TNAIIMA ranged from 0 (on 2 March 2020) to 1,037,129 (on 12 May 2020) and reached 851,602 on 30 June 2020.

**Figure 2:**
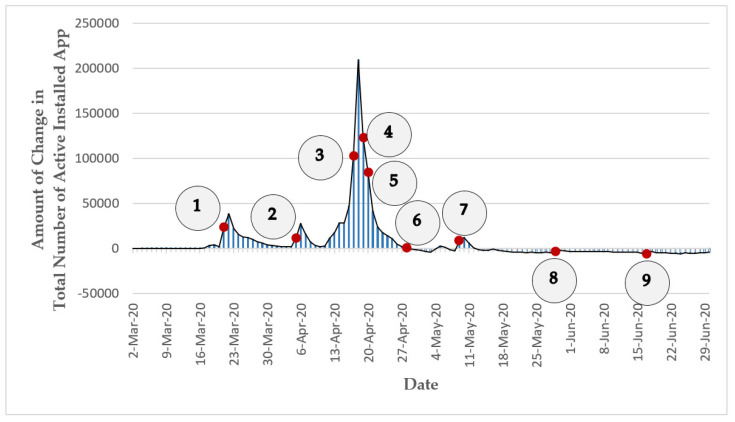
(1) Amount of change in the total number of active installed Mask apps (compared with the previous day) per day; (2) timeline of the identified television programs broadcasted by IRIB related to the Mask project and the endorsement, recommendation, or promotion of Mask project by the government (from 2 March to 30 June 2020). Notes: the figures for the amount of change in the total number of active installed Mask apps per day are limited to the installed instances of the Mask app downloaded from Cafe Bazaar Android marketplace; the numbering of the events is the same as that in Table 3.

Furthermore, the amount of change in the TNAIIMA (compared to the previous day) per day (i.e., the total number of new installations of the app on each day minus the total number of new uninstallations of the app on each day) was computed and illustrated in Figure 2. The amounts of change in the TNAIIMA in the study period ranged from −5755 (i.e., at least 5755 new uninstallations of the app compared to the previous day) on 24 June 2020 to +209,478 (i.e., at least 209,478 new installations of the app compared to the previous day) on 18 April 2020. The timeline of the identified events (see Table 3) was also depicted in Figure 1 and Figure 2.

Figure 2 shows that in most cases, a significant rise in the increment rate of TNAIIMA on the event day and its consecutive day (event day no. 1, 2, 3, 7 in Table 3) occurred after an event, or a significant drop in the reduction rate of TNAIIMA on the event day and/or its following day (event day no. 6, 8, 9 in Table 3) occurred compared to the previous day of the event. However, the magnitudes of these significant growths in increment rate or declines in the reduction rate of TNAIIMA are not the same. The significant escalation in the increment rate or the significant drop in the reduction rate of the TNAIIMA declines over the following few days of the event. Figure 2 also illustrates that the project experienced the sharpest rise in the increment rate of the TNAIIMA following event day no. 3 (sending the mass short message service (SMS) text messages by MOHME to all Iranian cell phones on 17 April 2020)—with at least 312,557 new installations on the event day and its consecutive day. Immediately after this intense wave of app installations, there were a series of television programs related to the Mask project and the endorsement and recommendation of the Mask project by the government on 19 and 20 April 2020 (event day no. 4 and 5). However, although there were considerable numbers of new installations of the app on and right after the days of event day no. 4 and 5 (at least 122,876, 84,615, and 41,088 new installations of the app on 19, 20, and 21 April 2020, respectively), they were less than the massive wave of new installations occurring after event day no. 3. It is expected that promotion, advertising, and marketing can potentially increase the total number of new installations and decrease the total of new uninstallations of the app each day. While more comprehensive research is still needed in this area, it seems that the cumulative effects of event day no. 3, 4, and 5 were (among) the main driving force(s) for the observed massive increase in the total number of new installations of the app between 17 and 21 April 2020 (at least 561,136 new installations during this period).

Some previous studies highlighted the positive impact of SMS text messaging on improving the uptake of public health interventions [182,183]. The SMS is also integral to emergency communications [184] for timely and fast dissemination of notifications for mitigating damage and reducing life loss. A previous study [185] showed that SMS, followed by television, have more comprehensive information dissemination capabilities than other information mediums, including microblogs and news portals in disaster pre-warning and recommendation. This study found that the SMS has a shorter delay time, higher coverage ratio, and speed of information dissemination among the other media mentioned above. A survey [186] in early April 2020 indicated that among the different organizations involved in COVID-19 crisis management in Iran, Iranians trust the healthcare system and MOHME of Iran the most, followed by the media (IRIB television service was identified as the most trusted and used media among the others in this study). The quantitative analysis is out of the scope of this study. While more data is required (that was not available for this research) and more comprehensive research is needed, it seems that these could (at least partially) explain the reason for attracting enormous numbers of people to the project in a short time after event day no. 3. According to another survey [187] in early April 2020 in Iran, 65.7% of survey participants identified the IRIB as their primary source of news about COVID-19, followed by other mediums including Telegram Messenger (8.3%), Instagram (7.2%), satellite television channels (6.3%), news websites (6.1%), friends and acquaintances (1.9%), and WhatsApp (1.4%). Moreover, 63.4% said they received their primary education about COVID-19 mainly from the IRIB. The equivalent figures for Telegram Messenger (7.2%), Instagram (7.2%), satellite television channels (3.9%), news websites (7.8%), friends and acquaintances (4.2%), and WhatsApp (2.8%) are significantly lower. These findings reveal the vast influence of the nation-wide media—IRIB (particularly the IRIB television service as the most popular service of IRIB)—in shaping public opinion about COVID-19-related issues and may explain the substantial impact of the television programs on attracting people to the project. Previous studies highlighted the importance of adequate and effective advertising and communication frequency in strengthening the previous information and reinforcing the audiences’ impression [188]. Therefore, while in general, it seems that the identified nine events served as the main driving forces behind the significant rise in the app’s uptake, it can be hypothesized that the app could achieve higher uptake levels if the frequency of the events (media coverages, endorsements, etc., by the government) in the aforementioned period was appropriately increased.

Overall, it can be stated that in Iran, the government’s role in raising public awareness about the COVID-related citizen science projects (such as the digital contact tracing project) was essential and irreplaceable. Additionally, the government plays a crucial role in informing people about the functionalities and requirements of these projects and their benefits for personal and public health and in encouraging citizens to adopt them due to its extensive and influential media power (mainly through the television service) and social capital [189] emerging from the health system (derived from the public trust in it). Therefore, the government should strengthen and expand its support for these projects, particularly for a participation-dependent voluntary project such as digital contact tracing, whose effectiveness and success is highly dependent on the broad participation of people in the project. Moreover, it should be noted that, similar to many other Iranian projects with citizen science characteristics, most of the participants who are involved in the Iranian COVID-19-related citizen science projects are not aware that they are participating in citizen science projects. Hence, raising the knowledge and understanding of the projects’ audience and participants about the concept of citizen science and its various benefits, alongside introducing these projects under the term of citizen science, may help to enhance the recruitment and retention of volunteers in these projects. Therefore, involving these considerations during the promotion, advertisement, and marketing for these projects is necessary. Furthermore, as mentioned earlier in Section 4, the previous studies revealed that moral, ethical, and religious values, beliefs, and motives are the main drivers of voluntary activities for emergency and crisis response in Iran. Traditionally, most of these activities have been carried out in the offline context. Consequently, the sphere of prosocial behavior in response to emergency and crisis circumstances has not been well expanded into cyberspace in public opinion, and the culture of online volunteering in this context has not been adequately formed in Iranian society. To this end, it is crucial to tightly connect the value and necessity of the contribution of citizens in online COVID-19-related citizen science projects (such as contact tracing programs) into the aforementioned moral, ethical, and religious values, beliefs, and motives. Therefore, since the media has a vast potential for promoting prosocial behavior and its drivers [190,191], the power of media should be exploited for promoting and recognizing moral, ethical, and religious merits of participating in COVID-19-related citizen science projects (including the contact tracing projects) in Iran that leads to fostering participation and long-term engagement of people in these projects. It is noteworthy that while generally, some mediums are more influential than others, the previous studies recommend the employment of a combination of different mediums (e.g., SMS, television, social media, news websites, print newspapers) for conveying the message and advertising, as this enhances the efficiency of information dissemination and marketing among various groups of audiences [185,192]. Thus, to raise public awareness and understanding about participation-dependent citizen science projects, it is crucial to take the distinct advantages of different mediums by exploiting combinations of communication channels.

##### 7.3.1.2. Recognition, Communication, and Feedback

Various intrinsic and extrinsic motives [193,194] could drive the engagement of people in citizen science projects [195,196]. Intrinsic motivation refers to engagement in an activity due to the achievement of internal rewards such as a sense of enjoyment, satisfaction, or pride. Extrinsic motivation refers to participation in an activity because of obtaining a separable outcome (i.e., attaining a specific external reward or avoiding a negative consequence) such as a score, money, or fame [193,194]. Since the creation of the mechanisms for fostering the intrinsic motivations of people for engagement in citizen science projects is difficult and not always possible, many project organizers prefer to rely on using extrinsic motives. In citizen science, people generously contribute their valuable time and effort for free; therefore, it is essential that all contributions of volunteers be recognized appropriately by the organizers of citizen science projects regardless of the level of their contributions [142,195]. The recognition of volunteers’ efforts in a meaningful and credible manner can also positively influence the long-term engagement of volunteers by extrinsically motivating them to continue their involvement in the project [168,197,198]. The form and amount of recognition should be appropriate to the nature of the project and tasks and consistent with the characteristics of volunteers such as their values, norms, and culture. Furthermore, the extrinsic incentives should be designed in a way that is not perceived as controlling an individual’s behavior and reducing their autonomy [199]. Otherwise, it may undermine the intrinsic motivation of participants, change how tasks are perceived by volunteers, reduce the self-image and social approval benefits, and tempt participants to play the system or cheat, resulting in drop-out and/or inaccurate outputs [200,201,202,203,204,205]. Recognition can happen in various ways in citizen science projects, such as writing a thank you message for all contributors, offering a certificate, promoting volunteers’ roles, or giving a gift, voucher, and monetary reward for high achievers [142,206]. The Iranian contact tracing app did not implement a concrete recognition system to cope with the drop-out problem. However, it seems that using the proper recognition and rewarding strategies in contact tracing projects such as the Mask app (and other similar citizen science projects in other areas) could assist in stimulating the app users’ motivations and overcome the problem of declining volunteer participation and retention over time. In this sense, different rewarding mechanisms (e.g., providing verbal appreciation, adding a badge to the volunteer’s online profile, offering a bonus or discount card, giving hygiene kits, and granting priority in receiving some services) should be carefully investigated to find the best solutions for providing incentives for long-term engagement of citizens in contact tracing projects and to ensure the trustworthiness of the data. It is noteworthy that the incentives for the contact tracing app users should be chosen in a way that does not cause injustice and consequently digital divide in the society (e.g., if the free hygiene kit is used as an external motive, it is required that the kits be also provided for the lower-income citizens besides the citizens who use a contact tracing app).

Citizen science projects require long-term outreach strategies and constant communication with the audiences via appropriate channels (e.g., email, SMS, blogs, discussion forums, and social networks) to sustain project engagement [132,168]. To this end, it is crucial to establish personalized communication with non-active members of the project, alongside frequent communication with the entire community [207]. As the level of involvement increases in a citizen science project from crowdsourcing to extreme citizen science (for more details, see: [208]), it is desirable to move from simple one-way communication to two-way communication (and more advanced levels of dialogic communication) in the project [132]. Like many other contact tracing projects, the Mask project suffered from the lack of a concrete communication plan for the retention of its users. However, it seems that defining and implementing a set of effective communication tactics is necessary for contact tracing projects such as Mask to cope with declining volunteer retention and drop-out over time.

The participants in the voluntary projects need to feel that what they are doing is useful and being appropriately used [160,209]. In this sense, providing continuous feedback on how a volunteer’s overall contribution or the entire outcome of a citizen science project helps citizen scientists understand how their contributions were used and how they impacted science, society, and the environment [132,210]. Prior experiments demonstrated that positive feedback could enhance the intrinsic motivation of participants compared to neutral or no feedback. [211]. Therefore, providing feedback plays a vital role in volunteer retention in citizen science projects [168]. No feedback was given to the Mask project participants. However, it is expected that giving feedback to participants of a contact tracing app about the number of traced infected and suspected cases of COVID-19 through the app could encourage them to continue their contribution to the project. The Mask project did not provide the general outcome of the project (e.g., the number of detected cases and true notifications) to the public. Nevertheless, it is believed that announcing the general outcome of a contact tracing project to the general public and reflecting the positive contribution of the project to the epidemiological investigation, epidemic control, and consequently the public health to the society accordingly may motivate more citizens to install and use the app.

### 7.4. Social Media as a Platform for Citizen Science Emergency Response Projects

The platform of an online citizen science project [212] may offer one or several services on the internet such as the gathering, processing, searching, discovery, and sharing of UGC, presenting UGC and other related data, describing tasks and guidelines, providing educational and technical materials and tools, making the potential target audiences aware of the volunteering opportunity in the project, registering the volunteers, and providing social networking, communication, and discussion features for the stakeholders of the project.

Social media is an integral part of daily life in many societies, with over 3.6 billion active users worldwide in 2020 [213]. Social media platforms provide an online interactive environment built on the foundation of the Web 2.0 paradigm through which individuals, communities, and organizations can connect and communicate together, can generate, share, present, and search content, can discuss their issues and opinions, and can learn about, explore, advertise, and promote things [214,215]. Designing and implementing a platform for a citizen science project from scratch enables the project’s organizers to personalize every aspect of the platform and the services it provides; however, this could be difficult, expensive, and time-consuming and might limit its visibility and adaptability. If developing a new platform for a citizen science project is not a feasible or optimal solution, an alternative option might be to use the customizable commercial/free citizen science platforms or deploy the existing non-specialized platforms such as social media platforms.

Previous studies have showed that social media can play an influential role in facilitating and shaping civic participation [216]. Social media allows for immediate interaction and communication with a large, diverse, and geographically dispersed audience. Most social media platforms are free to use and easy to start using. Moreover, many people are familiar with their features and functionalities. These platforms are usually built on robust, modern, scalable, fast, secure, and reliable infrastructures. Social media platforms can often effectively improve the awareness about and visibility of the activities. Consequently, the various features and functionalities of social media platforms can create many potential opportunities for providing the different services needed in citizen science projects.

Social media platforms have been mostly used as an advertising and promotion medium to recruit and retain volunteers in citizen science projects [217,218,219]. These platforms have also been deployed to facilitate interactive and real-time communication and discussion between citizens and other stakeholders of the citizen science projects, to provide feedback to volunteers, to post announcements and updates, to instruct volunteers, and to disseminate the projects’ results [219,220,221]. Relatively few citizen science programs have gone one step further and leveraged the capabilities of social media platforms for collecting the contributions of citizen scientists. For example, Soysal, et al. [222] used social media for crowdmapping of the urban red fox population. To this end, volunteers were asked to send sighting reports and/or photographs of the urban red foxes they observed with the observation location (either by sending a GPS coordinate or nearest cross streets) and time (as well as other optional information) to the organizers of this project via Facebook, Twitter, or Instagram. As another example, to record Italian dialects and document their linguistic variation, a citizen science project [223] developed a Telegram Bot (an automated software that runs inside the Telegram Messenger environment). This app, which runs inside Telegram Messenger, enables a volunteer to submit his/her voice recording and to enrich it with the associated location to his/her dialect (either by sending a GPS location or inputting the name of the place) into the project.

Both top-down citizen science projects (citizen science projects with top-down leadership) and bottom-up citizen science projects (citizen science projects with bottom-up leadership; i.e., collegial citizen science projects) can benefit from the various services that can be offered by social media platforms. Notably, the bottom-up citizen science projects with limited resources and budgets and the (top-down or bottom-up) citizen science projects that need to be launched urgently may take advantage of social media’s existing infrastructure to connect with the large numbers of potential audience and provide their services (e.g., collecting data, displaying or playing obtained data, discussing, advertising, and promoting) immediately and for free or at low cost. Two of the Iranian citizen science projects that were launched during the pandemic also took advantage of social media platforms for offering their services. Mask and CHIACD citizen science projects were both developed through a bottom-up approach by a group of volunteer citizens (without a top-down design and management of health and medical science professionals) in response to a biological crisis that impacted their society. The Mask project developed and launched its own platform for contact tracing in a short time after conceptualizing the project, relying on the profound expertise of its founders and its volunteer technical team in IT. Nevertheless, the Mask project employed a mixture of different social media platforms (Telegram Messenger, Instagram, Twitter, Soroush Messenger, Bale Messenger, and Aparat) for advertising and promoting itself in public and posting related news. Compared to the Mask project, the CHIACD project used social media’s capabilities more extensively—the strategy that allowed the organizers to eliminate additional workload or costs for developing the project’s platform. To this end, the CHIACD project leveraged the capacity of a combination of social media platforms (consisting of WeChat, Instagram, Aparat, Telegram, Twitter, and TritApp) to provide its various essential services such as UGC collection and raw data exchange, providing two-way communication, discussion, feedback, and output dissemination and presentation services. It is noteworthy that each social media platform has its own strengths and weaknesses; therefore, choosing the right social media platform or combination of social media platforms to provide the required services and functionalities for citizen science projects is pivotal to success.

## 8. Conclusions and Recommendation

This research shed light on the most significant online citizen science projects carried out to respond to the COVID-19 crisis in Iran. Moreover, this study attained and discussed some considerable insights and lessons learned from the failures and successes of these projects, enriching them by incorporating knowledge gained from other citizen science projects over the past years. Findings from this synthesis offer potentially valuable directions for the current and future citizen science projects that aim to respond to biological hazards such as the COVID-19 pandemic. This study highlighted some initial steps that need to be taken for capacity building for citizen science in Iran’s public sector and academic and research institutions. This research also argued that the provision of an effective response to an outbreak such as the COVID-19 crisis through citizen science requires building capacity and needs preparation for conducting citizen science projects in advance. Furthermore, this study discussed the influence of various organizational factors on the recruitment and retention of volunteers in COVID-19-related citizen science projects and mainly digital contact tracing programs. Remarkably, we highlighted the prominent role of project promotion, advertising, and marketing as well as recognition, communication, and feedback provision for the volunteers’ contributions on the participation and long-term engagement of participants of these projects that have been less focused on in the previous studies. This article also called attention to the high capacity of social media for providing a wide range of services (e.g., data acquisition, data presentation, discussion, advertisement, and promotion) for citizen science projects with limited resources and budgets or those required to be launched urgently. Limited/lack of access to up-to-date information about citizen science projects in Iran—particularly COVID-19-related citizen science projects—were two of the obstacles to doing this research. Moreover, the dearth of previous research on the various dimensions of Iranian COVID-19-related citizen science projects was among the main barriers to this research. However, we hope that this research can pave the way for future research in this direction.

COVID-19 has caused mental health problems among people worldwide, including people in Iran. Furthermore, schools and universities have been closed in Iran, and instruction has been switched to online as a part of measures to reduce the spread of COVID-19 in the country since late February 2020. This long-term disruption of in-class education has resulted in an education crisis in Iran, similar to many other countries. Engagement in citizen science projects could potentially improve the mental health and well-being of volunteers, and citizen science can serve as a beneficial educational tool for citizen scientists. Therefore, utilizing the potential capacity of relevant existing active Iranian citizen science projects such as those in the field of biodiversity monitoring, astronomy, and geographical mapping (while practicing social distancing) is recommended to reduce the aforementioned adverse consequences of COVID-19 in the country.

Citizen science has a broader implication in emergency response, one beyond this global pandemic. While citizen science has been studied extensively and practiced widely in some fields, relatively less contribution has been carried out on citizen science for disaster management. Currently, more than 4.6 billion people around the world actively use the internet. Moreover, the number of smartphone users worldwide today surpasses 3.5 billion and is to rise rapidly in the next few years. With the current penetration of the internet and smartphones, it is becoming much easier to engage people and communities in the disaster management process through citizen science. Higher engagement in these projects offers a vast range of opportunities, particularly for developing countries, where obtaining accurate and updated data and plans are always a significant challenge. The insights from this study can be used to overcome some existing barriers to operationalizing citizen science projects for disaster management and can be deployed to enhance the performance of these projects, particularly in developing countries.

## Figures and Tables

**Table 1:** The general specifications of five identified Iranian COVID-19-related online citizen science projects.

Project Name	Scientific Area	Number of Contributors/Registered Users	Least Expected Engagement Length for Task(s) Fulfillment	Technology for Data Collection/Data Exchange	Launch Date
CSASR	Epidemiological Monitoring	>12 million (by 3 April 2020)	One-time (if there is no change in the contributor’s health condition after the first contribution)	Dedicated Web App	4 March 2020
AC19	Epidemiological Monitoring	≈4 million (by 15 March 2020)	One-time (if there is no change in the contributor’s health condition after the first contribution)	Dedicated Web App/Dedicated Mobile App	3 March 2020
Mask	Epidemiological Monitoring	>1 million (by 21 April 2020)	Long-term	Dedicated Web App/Dedicated Mobile App	2 March 2020
Gharbalgar COVID-19	Artificial Intelligence	≈500 (by 28 April 2020)	One-time	Dedicated Web App/Dedicated Mobile App	10 April 2020
CHIACD	Community Health	≈300 (in early May 2020)	One-time/Short-term (depending on the type of the task)	Social Media	24 February 2020

**Table 2:** Some of the main characteristics of five identified Iranian COVID-19-related online citizen science projects.

Project Name	Outcome of Citizen Science Project	Leadership Approach	Stage(s) of Volunteers’ Involvement	Type of Project (Shirk et al. Typology)	Type of Project (Haklay Typology)
CSASR	Science-informed management/policy/response	Top-down: public sector-led	Single-stage: data gathering and basic processing/interpretation	Contributory	Crowdsourcing
AC19	Science-informed management/policy/response	Top-down: public sector-led	Single-stage: data gathering and basic processing/interpretation	Contributory	Crowdsourcing
Mask	Science-informed management/policy/response	Bottom-up: community-led	Multiple stages: problem definition, study design, research tools/methods design, data gathering and basic processing/interpretation, data analysis, and interpretation	Collegial	Extreme
Gharbalgar COVID-19	Scientific/research	Top-down: scientist-/researcher-led	Single-stage: data gathering and basic processing/interpretation	Contributory	Crowdsourcing
CHIACD	Science-informed management/policy/response	Bottom-up: community-led	Multiple stages: problem definition, study design, data gathering and basic processing/interpretation, presentation, and dissemination of results	Collegial	Extreme

**Table 3:** The list of identified television programs broadcasted by IRIB related to the Mask project and the endorsement, recommendation, or promotion of the Mask project by the government from 2 March to 30 June 2020.

Event Day No.	Date	Details
1	21 March 2020	Content: news report on Mask project [169] Host program (IRIB TV channel): a news program (IRIB TV1) Duration: 3:00 min Viewership level of host program: high
2	5 April 2020	Content: the minister of MOHME of Iran sent an official letter to the director-general of IRIB to introduce the Mask project and request for the showing of the animated advertisement videos of the project and the coverage of it in the IRIB’s programs [170]
		Content: news report on Mask project [171] Host program (IRIB TV channel): a news program (IRIB TV1) Duration: 2:16 min Viewership level of host program: low
		Content: news report on Mask project [171] Host program (IRIB TV channel): a news program (IRINN) Duration: 2:16 min Viewership level of host program: low
3	17 April 2020	Content: mass SMS message of MOHME of Iran to all cell phones nationwide with recommendation to install Mask app [172]
4	19 April 2020	Content: the president of Iran introduced the Mask app and its applications in monitoring and tracking the disease [173] Venue: in the briefing of National Task Force for Combating Coronavirus Disease weekly meeting (live broadcasted by IRINN)
		Content: news report on Mask project [174] Host program (IRIB TV channel): a news program (IRIB TV1) Duration: 2:54 min Viewership level of host program: high
		Content: talk show on Mask project [175] Host program (IRIB TV channel): an entrepreneurship and business program (IRIB TV1) Duration: 23:15 min Viewership level of host program: low
		Content: in-studio interview on Mask project [176] Host program (IRIB TV channel): a news program (IRIB TV1) Duration: 6:25 min Viewership level of host program: high
5	20 April 2020	Content: the spokesperson of the government of Iran announced the government’s (and the MOHME of Iran’s) support of the Mask app and recommended Iranians to use it [177] Venue: in the weekly press conference of the spokesperson of the government of Iran (live broadcasted by IRINN)
6	28 April 2020	Content: the spokesperson of MOHME of Iran announced that the Mask app is the only approved app by MOHME [178] Venue: in MOHME press conference
7	9 May 2020	Content: the deputy minister of MOHME of Iran recommended Iranians to install the Mask app [179] Venue: in MOHME press conference (live broadcasted by IRINN)
8	29 May 2020	Content: COVID-19 daily status report by the spokesperson of MOHME of Iran (including the recommendation to Iranians to use Mask app) [180] Host program (IRIB TV channel): a news program (IRINN) Duration: 00:36 min Viewership level of host program: low
9	17 June 2020	Content: news report on Mask project [181] Host program (IRIB TV channel): a news program (IRIB TV1) Duration: 3:17 min Viewership level of host program: high
